# Voltage–Time Transformation Model for Threshold Switching Spiking Neuron Based on Nucleation Theory

**DOI:** 10.3389/fnins.2022.868671

**Published:** 2022-04-13

**Authors:** Suk-Min Yap, I-Ting Wang, Ming-Hung Wu, Tuo-Hung Hou

**Affiliations:** Department of Electrical Engineering and Institute of Electronics, National Yang Ming Chiao Tung University, Hsinchu, Taiwan

**Keywords:** threshold switching selector, spiking neuron, nucleation theory, history-dependent, neuromorphic computing

## Abstract

In this study, we constructed a voltage–time transformation model (V–t Model) to predict and simulate the spiking behavior of threshold-switching selector-based neurons (TS neurons). The V–t Model combines the physical nucleation theory and the resistor–capacitor (RC) equivalent circuit and successfully depicts the history-dependent threshold voltage of TS selectors, which has not yet been modeled in TS neurons. Moreover, based on our model, we analyzed the currently reported TS devices, including ovonic threshold switching (OTS), insulator-metal transition, and silver- (Ag-) based selectors, and compared the behaviors of the predicted neurons. The results suggest that the OTS neuron is the most promising and potentially achieves the highest spike frequency of GHz and the lowest operating voltage and area overhead. The proposed V–t Model provides an engineering pathway toward the future development of TS neurons for neuromorphic computing applications.

## Introduction

With the increasing demand for massive data storage and processing, conventional computing systems based on the von-Neumann architecture have encountered their limitations. Frequent data transition between the separated processor and memory units makes conventional computation less efficient. Recently, emerging neuromorphic computing is regarded as the next-generation computing paradigm. Unlike the conventional von-Neumann-based computing system, brain-inspired neuromorphic computing not only provides energy-efficient computation with high parallelism but also shortens the latency of data transmission by realizing in-memory computing within crossbar memory arrays (Ielmini and Wong, 2018; Hua et al., 2019; Woo et al., 2019). In a neuromorphic computing system, an artificial synapse provides an adjustable and long-lasting weight value. In addition, an artificial neuron integrates and processes signals from synapses and then transmits the processed signals to the next neural layer as inputs. Both synapses and neurons have been extensively studied based on solid-state devices for neuromorphic hardware implementation (Lee et al., 2019a; Woo et al., 2019; Zhang et al., 2020). However, the conventional complementary metal-oxide semiconductor- (CMOS-)based neuron circuit occupies large chip areas because it requires a large number of transistors and capacitors for generating spike signals. In contrast, the neuron circuit area can be 10 times smaller by using novel devices, such as magnetoresistance memory (MRAM) (Wu et al., 2019, 2020; Liang et al., 2020), phase-change memory (PCM) (Tuma et al., 2016), and threshold switching (TS) selector (Park et al., 2016; Song et al., 2018; Grisafe et al., 2019; Hatem et al., 2019; Hua et al., 2019), which is beneficial for ultrahigh density neuromorphic computing applications (Liang et al., 2021).

Among several novel device-based neurons, threshold-switching selector-based neurons (TS neurons) are especially promising for ultra-high density neuromorphic architectures due to their simpler and smaller neuronal circuits (Liang et al., 2021). A circuit-level model solving Kirchhoff’s Law based on the resistor–capacitor (RC) equivalent circuit has been proposed to describe the behavior of TS neurons (RC Model) (Chen et al., 2016; Wang et al., 2020). However, the RC Model oversimplified the TS neuron by assuming constant switching behavior of the TS selector. Indeed, the switching dynamics of the real TS selector is affected by the external electric field, which can be explained using the nucleation theory (Karpov et al., 2008; Lee et al., 2020). Specifically, the way the external electric field is previously accumulated determines the device behavior, and we regard this time-dependent phenomenon as history dependence. Consequently, the TS voltage (*V*_th_) in the TS selector is history dependent rather than constant. In this study, aiming for constructing a more comprehensive and accurate neuron model, we proposed an improved voltage–time transformation model (V–t Model) on top of the original RC Model by considering the TS behavior both experimentally and theoretically.

In the following sections, we will first verify the spiking behavior of the TS neuron according to different synaptic weights. A silver- (Ag-)based TS selector was chosen to observe the switching dynamics and the history-dependent *V*_th_ of the device. Additionally, based on the nucleation theory, we will introduce a V–t transformation (V–t) equation to describe the variant *V*_th_ of the TS selector, and a V–t Model will be constructed. Furthermore, several types of TS neurons based on the reported TS selectors, including ovonic threshold switching (OTS) (Song et al., 2018; Hatem et al., 2019), insulator–metal transition (IMT) (Park et al., 2016), and Ag-based selectors (Grisafe et al., 2019; Hua et al., 2019), will be evaluated. The results suggest that the OTS neuron has the fastest spike frequency and a lower history-dependent *V*_th_. The V–t Model not only successfully depicts and predicts the characteristics of TS neurons, but it also provides a useful engineering guideline for future high-performance neuron circuits for neuromorphic computing applications.

## Experimental Details and Measurement Setup

### Ag-Based Threshold Switching Selector

**FIGURE 1:**
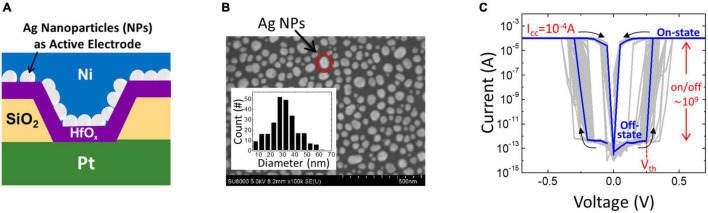
Silver- (Ag-) based threshold switching (TS) selector studied in this study. **(A)** The schematic structure showing the cross-section view of the Ag/hafnium oxide (HfO_x_)/Pt device. **(B)** The top-view scanning electron microscope (SEM) image of Ag nanoparticles (NPs) with an average diameter of 30 nm formed by 500°C rapid thermal annealing (RTA) for 5 min. The inset shows the size distribution plot of the Ag NPs. **(C)** DC current–voltage (I–V) characteristics of the device with a current compliance of 0.1 mA, showing the success of TS for 500 DC cycles.

In this study, an Ag/hafnium oxide (HfO_x_)/Pt TS selector was fabricated and investigated. The schematic illustration of the Ag-based TS selector is shown in Figure 1A. The Pt bottom layer was first deposited on a Ti/Si substrate using electron beam evaporation, followed by the silicon dioxide (SiO_2_) layer deposition using plasma-enhanced chemical vapor deposition. After the photolithography process, the reactive ion etching of SiO_2_ was applied to form a *via* contact with a diameter of 1 μm, which defines the effective device area. Then, the 4.5-nm-insulating HfO_x_ layer was deposited using atomic layer deposition. After that, 2-nm-thick Ag was deposited on the HfO_x_ layer using electron beam evaporation followed by rapid thermal annealing (RTA) at 500°C for 5 min to form Ag nanoparticles (NPs) as the active electrode. Finally, a 60-nm-thick Ni capping layer was deposited using electron beam deposition to prevent the oxidation of Ag NPs. Electrical measurements were performed using an Agilent B1500A and B1530A waveform generation/fast measurement unit at room temperature. Figure 1B shows the scanning electron microscope (SEM) image of Ag NPs. The size distribution of NPs is shown in the inset. Figure 1C shows the DC current-voltage (I-V) characteristics of the Ag/HfO_x_/Pt TS selector with 500 DC cycles of TS and a compliance current (*I*_cc_) of 0.1 mA. The device provides an extremely high on/off ratio (∼10^9^) and small *V*_th_ and hold voltage (*V*_hold_) for both positive and negative bias, showing typical behaviors of Ag-based TS selectors as reported in the literature (Yoo et al., 2017).

### Threshold Switching Neuron Circuit

**FIGURE 2:**
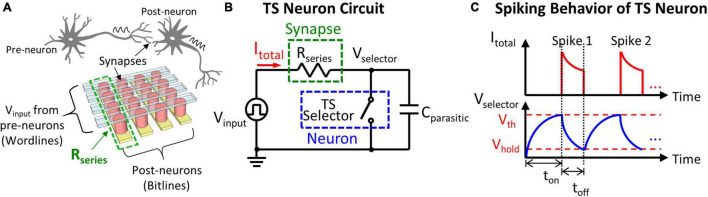
**(A)** Inspired by the biological neural network, neurons are connected with synapses through bit lines and word lines in the crossbar memory array. *V*_input_ received from the pre-neurons is applied on the word lines, and the post-neurons connected to bit lines generate output spikes according to the weight of synapses. **(B)** Equivalent circuit of the measurement setup where the synaptic devices along the same bit line (with a total resistance of *R*_series_) are connected in series with a TS selector as a neuron. A parasitic capacitor *C*_parasitic_ is considered and is connected in parallel with the TS neuron. **(C)** The evolution of *I*_total_ and *V*_selector_ shows the spiking behavior of the TS neuron when *V*_input_ is applied due to the charging and discharging of *C*_parasitic_. *t*_on_ and *t*_off_ define the required period of time for the TS selector to turned on and off in the neuron circuit, respectively.


To emulate neuromorphic hardware in which synapses and neurons are connected in the neural network (Figure 2A), the measurement setup adopted in this study is illustrated in Figure 2B. The effective resistor connected in series (*R*_series_) represents the total resistance of multiple synaptic devices in the synaptic array connecting in parallel to the same TS neuron. A parasitic capacitor (*C*_parasitic_) of the TS selector is exploited; therefore, no extra capacitor is needed for signal integration. The evolution of the total current (*I*_total_) flowing through *R*_series_ and the voltage across TS selector (*V*_selector_) is described in Figure 2C: when a constant input voltage (*V*_input_) is applied to the neuron circuit, most of the voltage initially drops across the TS selector in the off-state. Then, *V*_selector_ is gradually increased by charging *C*_parasitic_. Once *V*_selector_ reaches *V*_th_, the TS selector is switched to the on-state due to the formation of a volatile conducting filament, and an increase in *I*_total_ can be observed. However, *V*_selector_ drops right after the TS selector is switched to the on-state due to the discharge of *C*_parasitic_, and *I*_total_ starts to decrease. The TS selector returns to the off-state when *V*_selector_ reduces to *V*_hold_ because of the rupture of the volatile conducting filament. *t*_on_ and *t*_off_ define the required period of time for the selector to be turned on (*V*_selector_ to increase from *V*_hold_ to *V*_th_) and off (*V*_selector_ to decrease from *V*_th_ to *V*_hold_) in the neuron circuit, respectively. When the circuit is biased, a series of continuous current and voltage spikes are generated, and the spike frequency can be calculated as the number of spikes per second (Hz) accordingly. To fulfill the requirement of neural network applications, artificial neurons should be capable of generating different spike frequencies according to the weights of connected synapses, i.e., *R*_series_. In the RC Model (Chen et al., 2016; Wang et al., 2020), the *t*_on_ in the TS neuron circuit is obtained by

(1) $$t_{\text{on}}=-R_{\text{series}}\,C_{\text{series}}\times \text{ln}\,\left(\left|\frac{V_{\text{input}}-V_{\text{th}}}{V_{\text{input}}-V_{\text{hold}}}\right|\right)$$

**FIGURE 3:**
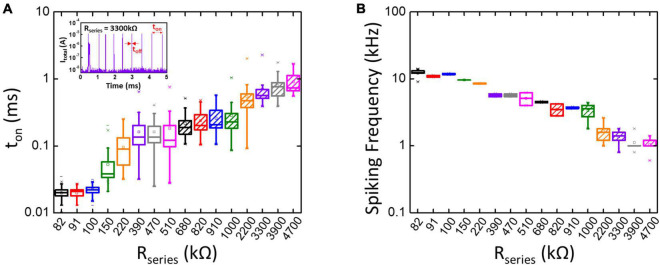
**(A)** The measured *t*_on_ increases with increasing *R*_series_ while the calculated spike frequency shown in panel **(B)** decreases with increasing *R*_series_ in the neuron circuit. The inset in panel **(A)** shows an example of the experimentally obtained spike current (*I*_total_) when *R*_series_ is 3,300 kΩ. The spike frequency is defined by the number of spikes per second (Hz). The spike frequency is approximately equal to the inverse of *t*_on_ when *R*_series_ is greater than 100 kΩ and *t*_on_ is much larger than *t*_off_.

In this study, we assume that the IR voltage drop on *R*_series_ is negligible when the TS selector is at its off-state due to the low leakage current (below pA in our case). Figure 3A illustrates the statistically measured *t*_on_ of the TS neuron when connecting to different *R*_series_, and the inset is an example of experimentally obtained current spikes (*I*_total_) when *R*_series_ is 3,300 kΩ. Figure 3B presents the calculated spike frequency, as shown in Figure 3A. The results indicate that, with the decrease of *R*_series_, *t*_on_ is decreased and the spike frequency is increased accordingly. However, the spike frequency cannot be further increased when *R*_series_ is < 100 kΩ. It is worth mentioning that *R*_series_ in the neuron circuit also acts as current compliance, where it controls the morphology and the size of conducting filaments in the TS selector (Chae et al., 2017). If *R*_series_ is too small, the filaments of extremely large size become non-volatile and cannot be ruptured even at *V*_hold_ = 0 V. Consequently, *t*_off_ increases and limits the spike frequency due to the difficult dissolution of large-size filaments in the TS selector. As a result, the resistance range of *R*_series_ requires careful adjustment (> 100 kΩ in our case) to prevent the dysfunction of neuron circuits. With a suitable range of *R*_series_ and with *t*_off_ being much smaller than *t*_on_, the spike frequency is the inverse of *t*_on_, thus proportional to the inverse of *R*_series_, i.e., the effective total conductance of the synaptic array.

## Results and Discussion

### History-Dependent *V*_th_ of the Threshold Switching Selector in Nueron Circuit

**FIGURE 4:**
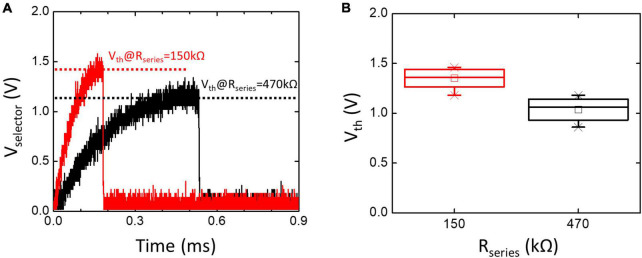
**(A)** The oscilloscope waveform of *V*_selector_ when the TS selector is connected with *R*_series_ of 150 and 470 kΩ and *V*_input_ = 2 V. Corresponding *V*_th_ is also indicated. **(B)** Statistically measured *V*_th_ increases with decreasing *R*_series_. Instead of remaining constant, the history-dependent *V*_th_ needs to be carefully considered in the TS neuron model.

An important assumption of the RC Model in Equation 1 is that the *V*_th_ of the TS selector is constant. Figure 4A compares the measured *V*_th_ captured by an oscilloscope with *R*_series_ of 150 and 470 kΩ, and the statistical results are indicated in Figure 4B. Instead of remaining constant, the *V*_th_ of the TS selector varies with *R*_series_. Different *R*_series_ modulate the charging rate of *V*_selector_ and give rise to the history-dependent *V*_th_. The naïve RC model does not consider the history-dependent *V*_th_ of the TS selector, thus reducing the accuracy and prediction capability of the neuron model.

### Voltage–Time Transformation Model

To include the characteristic of history-dependent *V*_th_ into the neuron model, the V–t Model is proposed. Starting from considering the switching dynamics of TS selectors when constant voltage stress (V_CVS_) is applied directly on the device, i.e., *V*_selector_ equals to *V*_CVS_. This is the case similar to Figure 2B but without the external *R*_series_. The time delay before turning on the selector (*t*_on_CVS_) is determined by the nucleation theory (Karpov et al., 2008; Lee et al., 2020):


(2) $$t_{\text{on\_CVS}}=\tau_{0}\,\exp\left(\frac{W_{0}\,\alpha^{\frac{3}{2}}\,E_{0}\,d}{k\,T\,V_{\text{CVS}}}\right)$$

where τ_0_ is the intrinsic time constant of the device, *W*_0_ is the nucleation barrier energy without electric field, α is a geometric factor of a nucleus, *E*_0_ is the voltage acceleration factor, *d* is the effective thickness of the insulating layer, *k* is Boltmann’s constant, and *T* is the ambient temperature. We define 𝔸 as a material-related constant at a fixed *T*, and (2) can be rewritten as


(3) $$𝔸=V_{\text{CVS}}\cdot \text{ln}\,\left(\frac{t_{\text{on\_CVS}}}{\tau_{0}}\right)=\frac{W_{0}\,\alpha^{\frac{3}{2}}\,E_{0}\,d}{k\,T}$$

where 𝔸 and τ_0_ can be obtained from fitting the measured *V*_CVS_ and *t*_on_CVS_. We assume 𝔸 remains constant when measuring the same device. Therefore, the V–t equation can be used to describe the transformation relation between any two arbitrary CVS voltages, *V*_CVS1_ and *V*_CVS2_, and their corresponding turn-on times, *t*_on_CVS1_ and *t*_on_CVS2_ as


(4) $$V_{\mathrm{CVS1}}\cdot \text{ln}\,\left(\frac{t_{\text{on}\,\_\,\mathrm{CVS1}}}{\tau_{0}}\right)=V_{\text{CVS}\,2}\cdot \text{ln}\,\left(\frac{t_{\text{on}\,\_\,\mathrm{CVS2}}}{\tau_{0}}\right)$$

**FIGURE 5:**
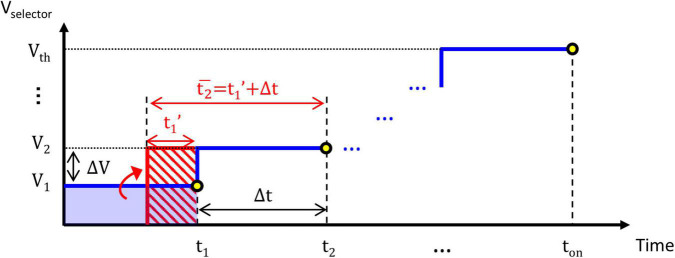
Time-varying *V*_selector_ in the neuron circuit is approximated using a finite number of constant voltage stress (CVS) steps from (*t*_1_, *V*_1_) to (*t*_2_, *V*_2_) and eventually to (*t*_on_, *V*_th_). Δ*V* and Δ*t* determine the voltage and time intervals, respectively. Based on the proposed V–t Model, the transformed (*t*_1_, *V*_2_) step indicated by the red-dashed rectangle is equivalent to the (*t*_1_, *V*_1_) step indicated by the blue-filled rectangle. The new $\bar{{\text{t}}_{2}}$ of *V*_2_ is now *t*′_1_+Δ*t*, which includes the history effect of the previously accumulated (*t*_1_, *V*_1_) step.


When connecting the TS selector with *R*_series_ to form a complete TS neuron circuit, as shown in Figure 2B, *V*_selector_ becomes time-varying according to the RC equivalent circuit. The time-varying *V*_selector_ could be approximated using a finite number of CVS steps as depicted in Figure 5, which increase from (*t*_1_, *V*_1_) to (*t*_2_, *V*_2_) and eventually to (*t*_on_, *V*_th_) indicated by the blue line. Δ*V* and Δ*t* are the voltage and time intervals, respectively. A similar conversion between CVS and ramp voltage stress has been reported and validated in resistive switching memory devices (Luo et al., 2013). As indicated in Figure 5, the stress effect of the (*t*_1_, *V*_1_) step indicated by the blue-filled rectangle on the device is transformed to that of an equivalent (*t*′_1_, *V*_2_) step indicated by the red dashed rectangle based on (4), *t*′_1_ is therefore expressed as:

(5) $$t_{1}^{\prime }=\exp\left[\frac{V_{1}}{V_{2}}\cdot \text{ln}\,\left(\frac{t_{1}}{\tau_{0}}\right)+\text{ln}\,\left(\tau_{0}\right)\right]$$

A new equivalent CVS step of ($\bar{{\text{t}}_{2}}$, *V*_2_) with an equivalent stress time of $\bar{{\text{t}}_{2}}$ = *t*′_1_ + Δ*t* at *V*_2_ includes the history effect of the previous (*V*_1_, *t*_1_) step. This equivalent stress time is accumulated until it reaches the *t*_on_CVS_ at the stop voltage, i.e., *V*_th_, which could be calculated by Equation 2. Under these circumstances, *V*_th_ becomes history-dependent and is affected by the RC charging process and *R*_series_. The larger *R*_series_, the lower *V*_th_, and longer *t*_on_.

**FIGURE 6:**
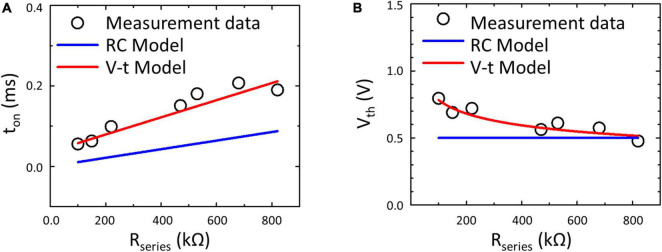
Measurement data and simulated results of **(A)** *t*_on_ and **(B)** *V*_th_ of the TS neuron with different *R*_series_. The results suggest a more accurate prediction based on the V–t Model than the RC Model (Chen et al., 2016; Wang et al., 2020) by considering the history-dependent *V*_th_ of the TS selector.

To confirm the feasibility of the V–t Model on the prediction of the TS neuron behavior, the simulation results obtained from the RC Model (Chen et al., 2016; Wang et al., 2020) and the proposed V–t Model are compared in Figures 6A,B with the measurement. The RC Model only describes the RC behavior of the neuron circuit with a constant *V*_th_ of the TS selector, therefore it not only underestimates *t*_on_ but also fails to depict the history-dependent *V*_th_ of the TS selector. In contrast, the proposed V–t Model predicted well *t*_on_ and *V*_th_ of the TS selector under different *R*_series_.

### Prediction of Threshold Switching Neuron Performance Based on V–t Model

**FIGURE 7:**
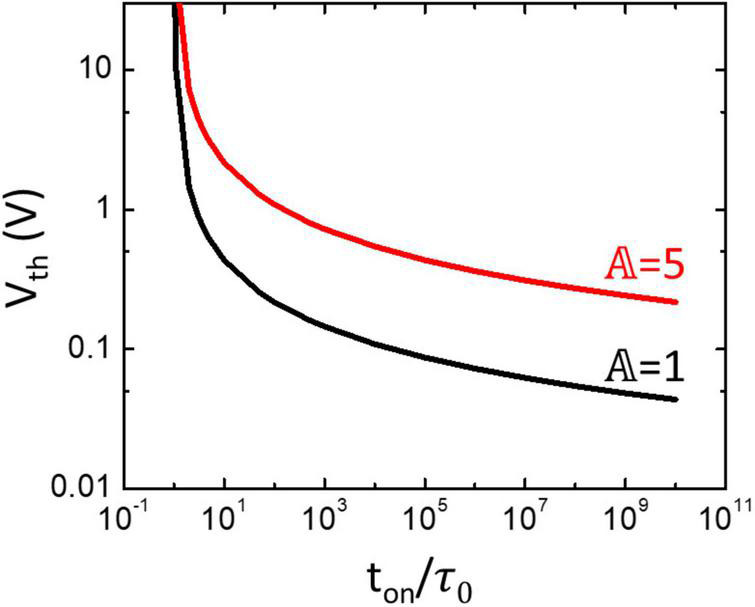
Relation between *t*_on_, τ_0_, 𝔸, and *V*_th_ of TS selectors based on the nucleation theory in Equation 2.

In this section, we explored the impact of the TS selector on TS neurons, and the effect of *t*_on_ and 𝔸 on *V*_th_ can be predicted based on Equation 2. As shown in Figure 7, when *t*_on_ approaches τ_0_, the voltage required for nucleation (*V*_th_) approaches infinity. In addition, the TS selector with larger 𝔸 requires a higher *V*_th_ to be turned on. These results indicated that, under the same *t*_on_, the TS selector with larger τ_0_ and 𝔸 needs a higher applied voltage than the one with smaller τ_0_ and 𝔸. However, the required high applied voltage is not favorable because it not only may result in an irreversible breakdown of the device but also may increase the difficulty of circuit integration. Therefore, the TS selector with larger τ_0_ and 𝔸 may require an additional external integration capacitor to maintain a reasonable *V*_th_, which on the other hand increases the circuit footprint and *t*_on_ and decreases the spike frequency. The energy consumption per spike of the neuron circuit could also increase due to slow spiking (Liang et al., 2021).

**FIGURE 8:**
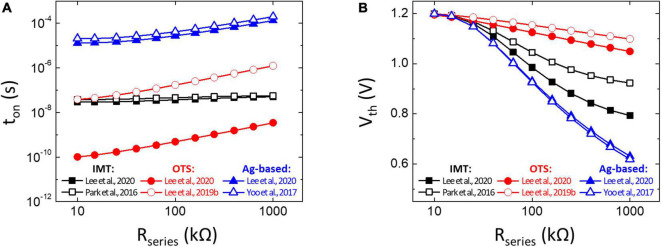
V**–**t Model prediction on **(A)** *t*_on_ and **(B)** *V*_th_ of the neuron circuit based on insulator–metal transition (IMT) (Park et al., 2016; Lee et al., 2020), ovonic threshold switching (OTS) (Lee et al., 2019b,^2020^), and Ag-based (Yoo et al., 2017; Lee et al., 2020) selectors. *R*_series_ is assumed to range from 10 kΩ to 1 MΩ. The value of an integrated capacitor in each condition is listed in Table 1.

Table 1 lists the reported parameters of τ_0_ and 𝔸 of different TS devices (Park et al., 2016; Yoo et al., 2017; Lee et al., 2019b,^2020^), and the simulated *t*_on_ and *V*_th_ of the neuron circuit based on the V–t Model are indicated in Figure 8. In this study, the *R*_series_ is given from 10 to 1,000 kΩ. The value of the integration capacitor in the neuron circuit is adjusted to keep the maximum *V*_th_ below 1.2 V at *R*_series_ = 10 kΩ, and the adopted capacitance corresponding to each TS device is also given. Among IMT, OTS, and Ag-based TS devices, the OTS neuron matched with the lowest capacitance is the most favorable for reducing the neuron circuit area. It is noted that the minimal integration capacitor is limited by the parasitic capacitor of the TS selector itself. As a result, the device area scaling would be necessary to achieve a low enough capacitance value. Moreover, the simulated *t*_on_ in Figure 8A shows that the OTS neuron is capable of achieving GHz-level spike frequency due to its extremely small τ_0_ (10 ^– 21^s), even though its 𝔸 is larger. Furthermore, in Figure 8B, the OTS selector with the smallest τ_0_ results in a large *t*_on_/τ_0_; therefore, the *V*_th_ is less history dependent. The OTS selector shows promising potential not only in generating high spike frequency but also consuming less area and energy in the neuron circuit.

**TABLE 1:** Key parameters extracted from the reported threshold switching (TS) selectors.

TS selector type	𝔸 (V⋅s)	τ _0_ (s)	Capacitor (F)*
IMT (Lee et al., 2020)	1.29	10^–8^	6 × 10^–13^
IMT (Park et al., 2016)	1.602	10^–8^	7 × 10^–13^
OTS (Lee et al., 2020)	30.28	10^–21^	2 × 10^–15^
OTS (Lee et al., 2019b)	45.78	10^–24^	6 × 10^–16^
Ag-based (Lee et al., 2020)	3.09	10^–6^	3.25 × 10^–10^
Ag-based (Yoo et al., 2017)	2.92	10^–6^	2.95 × 10^–10^

*The value of an integrated capacitor in the neuron circuit is adjusted to keep the maximum V_th_ below 1.2 V at R_series_ = 10 kΩ.

## Conclusion

In this study, a V–t Model is successfully constructed to simulate the spiking behavior of TS neurons according to the synaptic weight of connected synapses. By considering the history-dependent *V*_th_ of the TS selector based on the nucleation theory, the proposed V–t Model is in good agreement with the measurement results and provides more accurate prediction compared to the conventional RC Model. Moreover, the behavior of TS neurons based on different TS devices, including IMT, OTS, and Ag-based selectors, are simulated and compared using the proposed V–t Model. The results show that the OTS selector matched with the lowest capacitance that is the most favorable for reducing the circuit area overhead. Moreover, the OTS selector with the lowest τ_0_ and *t*_on_ not only achieves less history-dependent *V*_th_ but also realizes a high-speed neuron with GHz-level spike frequency. The proposed V–t model provides a useful engineering pathway toward the future development of TS neurons.

## Data Availability Statement

The raw data supporting the conclusions of this article will be made available by the authors, without undue reservation.

## Author Contributions

S-MY fabricated the device. S-MY and M-HW performed data analysis. S-MY, I-TW, M-HW, and T-HH contributed to the conception and discussion of the study. S-MY, I-TW, and T-HH drafted manuscript. All authors contributed to the article and approved the submitted version.

## Conflict of Interest

The authors declare that the research was conducted in the absence of any commercial or financial relationships that could be construed as a potential conflict of interest.

## Publisher’s Note

All claims expressed in this article are solely those of the authors and do not necessarily represent those of their affiliated organizations, or those of the publisher, the editors and the reviewers. Any product that may be evaluated in this article, or claim that may be made by its manufacturer, is not guaranteed or endorsed by the publisher.
